# Job stress and job involvement among tertiary interns: the buffering role of perceived coworker support

**DOI:** 10.1016/j.heliyon.2022.e10414

**Published:** 2022-08-30

**Authors:** Edem M. Azila-Gbettor, Ernestina Atsu, Abigail Nana Konadu Quarshie

**Affiliations:** Department of Management Sciences, Ho Technical University, Ghana

**Keywords:** Job stress, Job involvement, Perceived co-worker support, Internships, University students

## Abstract

Job stress is a major challenge for both organisations and individuals. Considerable studies have highlighted the incalculable and detrimental influence of job stress on employees' job behaviour, including job involvement. Additionally, studies devoted to job stress and job involvement are skewed towards formal settings. This study investigates the moderating effect of perceived co-worker support on the influence of job stress on job involvement among student interns from public and private institutions. A total of 452 students took part in the research by completing an online self-reported questionnaire. The respondents were chosen using a stratified sampling method. The data was processed and analysed using IBM SPSS version 24 and SEM PLS, respectively. Results reveal that job stress experienced by interns negatively predicts job involvement whilst perceived co-worker support positively moderates the relationship between intern’s job stress and job involvement. The findings suggest organisations must build a supportive work culture in the work environment in order to facilitates interns' job involvement.

## 1 Introduction

Globally, higher educational institutions have integrated internship programmes as a requirement for students' graduation (Rothman and Sisman, 2016). Internship is considered as a means of student’s familiarising with the work environment and a necessary conduit of student’s development process through the acquisition of practical knowledge (Liu et al., 2011; Weible, 2010). Studies have shown that internship programmes enhance students' professionalism, thereby making them active, independent, and effective in solving problems as well as prospects for employment (Anjum, 2020; Busby and Gibson, 2010). Internships are excellent sources to enrich students' real work experience and enhance their ability to work in teams (Raskin, 1994; Ronnestad and Skovholt, 1993). Inspite of the several benefits associated with internship programmes (Ebner et al., 2021; Knouse and Fontenot, 2008; Prescott et al., 2021), results of several studies revealed interns experience several challenges including limited learning opportunities, rigid rules, negative attitudes of supervisors, unfriendly working environments, work slave and job stress (Divine et al., 2007; Johari and Yahya, 2019; Li, 2018; Thornton et al., 2019).

Job stress is considered a negative experience and may cause an individual to leave their job or not accept job offers in their chosen discipline (Johari and Yahya, 2019). In the higher education setting, studies have shown that the level of stress among interns is high (Abdulghani et al., 2014; Elsaid et al., 2019; Nolan and Ryan, 2008), with much of the stress linked to work demands. Some scholars have highlighted deficiency in knowledge and skills, no remuneration, monotonous work, transport challenges, demands from several superiors, and long working hours as key factors leading to stress among student interns (Elsaid et al., 2019; Mensah et al., 2021). This notwithstanding, much work on job stress among interns has focused on health students (Abdulghani et al., 2014; Elsaid et al., 2019; Nolan and Ryan, 2008; Rabei et al., 2020), with little work on other disciplines among university students (Bam et al., 2014; Mensah et al., 2021). Besides, job stress has been found to negatively influence employees' job involvement (Johari and Yahya, 2019; Li, 2018; Walia and Narang, 2015). Stevenson and Harper (2006) found stress to negatively impact students' learning experiences. A review of existing literature suggests that even though studies on stress among university students have received considerable attention (Hannan et al., 2018; Mensah et al., 2021; Zolhavarieh et al., 2020), the link between job stress and job involvement of interns in the higher education environment remains unexplored.

In another vein, studies have demonstrated the positive influence of perceived co-workers' support on the negative association between stress and several outcome variables at both organizational and individual level (Ahmad et al., 2019; Karatepe, 2012; Shin et al., 2021). Inspite of the significant contribution of perceived co-worker support, there is a paucity of evidence on the influence of perceived co-worker support on the link between job stress and job involvement among interns in the context of higher education. Therefore, this study seeks to explore how perceived co-worker support may impact the relationship between intern’s job stress and job involvement.

The study makes the following contributions to the higher education literature. First, the study expands the literature on job stress and job involvement by focusing on the higher education environment in sub-Saharan Africa. Second, the study extends the existing literature by examining the moderating effect of perceived co-worker support on job stress and job involvement.

## 2 Literature review

### 2.1 Job stress

Though there is a wide range of opinions on what constitutes stress (Orlans, 1991), a commonly recognised definition is “one of interaction between the situation and the individual” (Michie, 2002, p. 67). In general, job-related stress has been theorized in terms of inconsistency between employee capability and job requirements or organizational demand (Pediwal, 2011). Milutinović et al. (2012, p. 171) opined that “job-related stress starts when fulfilments of the working environment overpower the capacities of workers to deal with them.” Milbourne and Wilkinson (2015) conceptualised job stress as mystical exhaustion triggered by the exposure of individuals to heavy work when individuals feel tired and deflated. The physical and mental exhaustion at work, according to Song et al. (2015), would result in a lack of worker enthusiasm, high frustration, nervousness, and even insomnia, headaches, anxiety, and depression. Cavanaugh et al. (2000) classified stress based on its causes as either “challenge-related stress” or “hindrance-related stress”. Challenge-related stress is caused by work demands, including workloads, time, and the scope of work at the workplace, while hindrance-related stress occurs because of the conditions of work, such as organisational politics, job insecurity, formalities at work, the ambiguity of roles, and tall hierarchical structures. In this study, internship stress is defined as a psychological or physical demand reaction to job expectations during a student’s internship (Mensah et al., 2021).

Several notable studies in the field of sciences have shown interns to experience stress due to poor coping with uncertainty, sense of responsibility, and negative interpersonal experiences (Bradshaw et al., 2018; Liu et al., 2016; Sun et al., 2008). Stress has been found to negatively affect behaviour and interpersonal relationships within an organisation (Chen and Silverthrone, 2008; Tourigny et al., 2013). Among nursing interns, Rabei et al. (2020) found stress to increase the phenomenon of absenteeism, turnover, and impaired effective functioning of individuals. In a comparative study between marketing and hospitality students based on 285 respondents, Mensah et al. (2021) found stress to reduce interns' satisfaction levels and increase their turnover intentions. Similarly, Khairuddin (2017) found stress to limit the work performance of 250 interns in Malaysia. In an earlier study by Gordon et al. (1986), interns who experienced stress were equally found to experience changes in four mood factors including, tension-anxiety, anger-hostility, fatigue-inertia and vigor-activity. Internship stress was found to lead to the inability to learn (Abdulghani et al., 2014) and sleep disturbances among nursing interns (Rabei et al., 2020).

### 2.2 Job involvement

The concept of job involvement was conceptualised by Lodahl and Kejner (1965) and defined by Kanungo (1982) as “one’s psychological identification with the job”. Paullay et al. (1994) provided a much-detailed definition of job involvement to include “the degree to which one is cognitively preoccupied with, engaged in, and concerned with one’s present job”. Studies have established that employees who are actively involved in their jobs exert all the necessary efforts to ensure that the goals of the organisation are achieved with low average turnover (Kahn, 1990). Highly involved employees are more likely to perform their duties with confidence and independence (Chen and Chiu, 2009, which has a direct impact on job performance (Moen et al., 2016). Further, studies have revealed that job involvement promotes career commitment (Ahmed, 2019) and organizational commitment (Jyoti et al., 2020). Chen and Chiu (2009) identify individuals with high job involvement with independent character and self-confidence.

### 2.3 Job stress and job involvement

Bijwaard and Wang (2016) opined that stress has both psychological and physiological influence on employees at work, especially when individuals' abilities cannot match their corresponding expectations. Evidence from studies has confirmed a negative relationship between job stress and job involvement (Adekeye et al., 2017; Demir, 2018; Li, 2018; Walia and Narang, 2015). For example, in a study of 117 IT professionals in India, Walia and Narang (2015) found job stress to correlate negatively with job involvement. In another study, Adekeye et al. (2017) confirmed a negative relationship between job stress and job involvement among 180 employees drawn from private and public sector employees. In a recent study, Qureshi et al. (2019) found job stress negatively affected the job involvement of 827 police officers.

The negative association between job stress and job involvement can be explained using a job demand/strain model (Karasek, 1979). Karasek (1979, p. 291) defined job demands as “the psychological stressors involved in accomplishing the workload, stressors related to unexpected tasks, and stressors of job-related personal conflict”. Lambert et al. (2013a, 2013b), likewise Wong and Laschinger (2015) suggest that these stressors in the work environment increase employees' negative outcomes. Similarly, Sargent and Terry (2000) opined that these excessive job demands result in individuals having minimum control over their work, heightening their psychological strain, which, in turn, raises the level of stress from the job. Anecdotal evidence suggests interns faced high job demand in the form of role ambiguity, conflict, overload and task receptiveness. We therefore argue that these pressing factors may result in interns' frustration and psychological strain and reduce their job involvement (Lambert et al., 2016; Lambert et al., 2013; Schaufeli and Taris, 2014). Based on the following, it is hypothesised that:

H1 *Job stress negatively predict intern’s job involvement of interns.*

### 2.4 Moderating effect of perceived co-worker support

Perceived co-worker support constitutes a component of social support (Karatepe, 2012) and is described as a “social resource” (Mazzetti et al., 2016). Perceived co-worker support is defined as “the extent to which one’s co-workers are helpful, can be relied upon in times of need, and are receptive to work-related problems” (Menguc and Boichuk, 2012, p. 1360). Perceived co-worker support has been found to diminish the negative feelings employees have about the functions performed in their organisation because of the invigoration obtained from the perceived values of the relationship (De Clercq et al., 2020). The expert knowledge that co-workers share, instils confidence in employees, allowing them to overcome any knowledge gaps in the job at hand and successfully complete their work tasks (Cho and Johanson, 2008). McCalister et al. (2006), likewise, Park et al. (2016) in their studies disclose that co-worker support influences job satisfaction among employees as well as serves as a positive effect on the job environment. Co-workers also serve as confidants and lighten workloads (López et al., 2019).

The individual’s work environment, including the availability of materials and resources, is considered very important in determining their level of job stress and how they can meet the demands of the work. Scholars have argued that work-base support, such as organisational, supervisory, and co-worker support, might differently qualify the relationship between antecedent variables and employee outcomes (Yang et al., 2020; Kim et al., 2017; Montani et al., 2012). In this study, we selected perceived co-worker support as a boundary condition in examining the relationship between stress and job involvement of interns. Academics posit that co-workers serve as a positive influence on employees' job environments (McCalister et al., 2006; Park et al., 2016). Consequently, we argue that the extent to which job stress may affect interns' job involvement is contingent on their co-workers. Thus, in job environments where co-worker support exists, interns benefit from their knowledge and expertise (Zhou and George, 2001), which may likely reduce their job stress (De Clercq et al., 2020) and increase their job involvement. This study hypothesises that perceived co-worker support will strengthen the relationship between an intern’s job stress and job involvement.

The moderating effect of co-worker support is rooted in the social capital theory (SCT). The fundamental idea of SCT is that individuals acquire tangible and intangible resources through social interactions and relationships with others at the individual, group, and organisational levels (Bourdieu, 1986; Putnam 2000). Proponents of SCT posit that social interactions serve as social networks of value which is beneficial to those who participate in them (Beames and Atencio 2008). These networks make it easier for individuals and organisations to provide valuable psychosocial resources to support colleagues (Guo et al., 2019). We contend that co-worker support is an imperative resource to mitigate the negative effect of job stress on the job involvement of interns. Thus, when interns are provided with support, the negative link between stress and job involvement will be lessened. Based on the foregoing, we infer the following hypothesis:

H2 Perceived co-worker support positively moderates the relationship between intern's job stress and job involvement.

**Figure 1:**
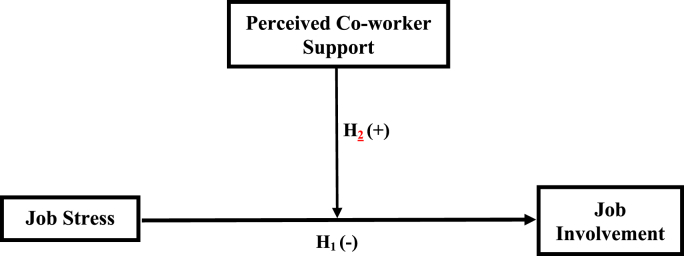
Moderation model of job stress, job involvement and perceived coworker support.

The model below illustrates the expected relationship between the three variables (see Figure 1).

## 3 Methods

### 3.1 Participants and procedure

The study’s participants were full-time business students at a Ghanaian technical university. The research sample size was determined to be 520 based on a population of 8,000 (Krejcie and Morgan, 1970). The respondents were chosen using a stratified sampling method. The sample frame was generated using student registration lists obtained from the institution’s registry. The Google web platform was used to create the questions. Students were only allowed one attempt at the question to avoid repeated entries. The questionnaires were administered to the respondents using either email or WhatsApp after they were contacted on the phone for their preferred option. The data was gathered between July and September 2020. Respondents' consent was sought for prior to sending out the questionnaires. Furthermore, participants were assured of confidentiality and privacy of data gathered. The study was approved by the university's Research and Ethics Review Committee, and it fulfilled the provisions and principles of the Declaration of Helsinki for research on human subject.

Out of 520 questionnaires administered, 452 were found to contain adequate information needed for data analysis, yielding an overall response rate of 87 percent. Of the 452 respondents, 67.5% were females and 32.5% were males. Majority of the respondents were between 21 and 25 years old (61.7%), and single (94.1%). Most of the respondents were in their third year (66.8%) and did their internship in public sector organisations (67.9%) (Table 1). Sample public organisations include universities, district and municipal assemblies, national/regional/district health insurance authorities, and hospitals, etc., while examples of public organisations include commercial banks, supermarkets, private hospitals, etc. The age and marital status distribution of respondents are typical of Ghanaian university students (Azila-Gbettor et al., 2020, 2022, 2021; Mensah and Azila-Gbettor, 2018; Mensah et al., 2021).

**Table 1:** Demographic profile of sample.

Characteristic		Frequency	Percent
Gender	Male	147	32.5
	Female	305	67.5
Age	≤20 yrs.	31	6.9
	21–25 yrs.	279	61.7
	≥26 yrs.	142	31.4
Marital Status	Married	39	8.6
	Single	413	91.4
Year	Level 200	150	33.2
	Level 300	302	66.8
Place of Internship	Private	145	31.9
	Public	307	67.9

### 3.2 Measures

The questionnaire was divided into 2 sections. Section A solicited information on respondents' profiles such as age, marital status, gender, age, programme of study, place of attachment, and year of study. Section B focused on the variables examined in the study, including job involvement, perceived co-worker support, and job stress. A total of 27 items were adopted for use from self-reported validated measures (Table 2). All the scales were originally in English. The survey was initially piloted using 100 students from a teacher training college. The reported Cronbach alpha values after the pilot study are 0.879 (job stress), 0.712 (job involvement), and 0.847 (perceived co-worker support).

**Table 2:** Sources of measures of concepts.

Latent Construct	No. of Items	Source	Range of Scale
Job involvement	4	Singh and Gupta (2015)	1 (*strongly agree*) to 5 (*strongly disagree*)
Perceived co-worker support	8	Karasek et al. (1998)	1 (*strongly agree*) to 5 (*strongly disagree*)
Job stress	15	Wu et al. (2018)	1 (*strongly agree*) to 5 (*strongly disagree*)

Information regarding the scales is presented in Table 2. *Job stress* was measured using a 15-item scale developed by Wu et al. (2018). Sample item includes “*My job has not been clearly explained and explained*”. *Job involvement* was measured using a 4-item scale developed by Singh and Gupta (2015). Sample item includes “*My job during attachment is the most important part of my life*”. Finally, perceived *co-worker* support was measured using a 8-item scale developed by Karasek et al. (1998). Sample items includes “*Workers at my place of attachment took over my work/task anytime I was tired*”. All items were measured on a 5-point Likert scale ranging from 1 (*strongly agree*) to 5 (*strongly disagree*).

### 3.3 Analytical approach

IBM SPSS statistical version 24.0 was used to process the data. The characteristics of the respondents were examined using descriptive statistics. The hypotheses were verified using Partial Least Square-Based Structural Equation Modelling (PLS-SEM). PLS-SEM was chosen because of its capacity to estimate causal links across all latent components concurrently while also dealing with measurement errors in the structural model (Farooq, 2016; Hair et al., 2017). The technique of Kock (2015) was utilised to assess common method bias. According to the findings (Table 3), the all-factor level Variance Inflation Factors (VIFs) obtained from a multicollinearity test are less than 3.3, suggesting an absence of common method bias. Finally, a simple slope analysis was conducted to facilitate the interpretation of the moderation.

**Table 3:** Factor loadings, VIF, validity and reliability of latent constructs.

Constructs and Items	Loadings	VIF	CR	CA	AVE
Job Stress (JS)			0.938	0.928	0.559
JS_1_	0.709	2.038			
JS_2_	0.789	2.798			
JS_3_	0.768	2.495			
JS_4_	0.764	2.317			
JS_5_	0.831	2.859			
JS_6_	0.802	2.977			
JS_7_	0.809	2.079			
JS_8_	0.721	2.090			
JS_11_	0.832	1.734			
JS_14_	0.819	1.514			
JS_15_	0.686	2.858			
Job Involvement (JI)			0.848	0.740	0.735
JI_1_	0.853	1.285			
JI_3_	0.862	1.285			
JI_4_	0.878	1.295			
Perceived Co-worker Support (PCS)			0.953	0.941	0.773
PCS_1_	0.889	1.802			
PCS _2_	0.890	1.812			
PCS _4_	0.810	2.352			
PCS _5_	0.913	1.172			
PCS _6_	0.877	1.188			
PCS _7_	0.891	2.542			

## 4 Results

### 4.1 Measurement model assessment

The measurement model's quality was evaluated using the validity and reliability of coefficients of latent constructs. The tests of reliability and validity were conducted and confirmed by iteratively observing the factor loadings, and items of latent constructs that did not meet the threshold of 0.7 were removed. For example, one, four, and two indicators of job involvement, job stress, and perceived co-worker support were respectively deleted. As reported in Table 3, the model is assumed to be suitable for structural analysis based on the results of the latent constructs (Hair et al., 2017). For instance, the coefficients of Composite Reliability (CR) ranged from 0.848 to 0.953, which exceeds the suggested limit of 0.70 (Bagozzi and Yi, 1988). Besides, the Cronbach alpha (CA) coefficients ranged from 0.740 to 0.941, which were higher than the recommended upper limit of 0.7 (Nunnally, 1978). In addition, the average variance extracted (AVE) values for all variables surpassed 0.50, ranging from 0.559 to 0.773, confirming the model’s latent variables' convergence validity and reliability (Hair et al., 2014).

Fornell and Larcker (1981) and HTMT criteria were used to test the model’s discriminant validity (Henseler et al., 2015). The square root of all constructs' AVEs in the matrix diagonal is larger than the related correlations in the matching columns and rows, as shown in Table 4, indicating the reflective model’s quality (Hair et al., 2013). For example, the square root of the AVE for job stress (0.747) is larger than the equivalent column (−0.208) and row correlations (−0.201). As a result, the three latent constructs assessed in the study are distinct, suggesting that the measured constructs are of high quality. All the correlations (Table 4) for the HTMT criteria for measuring discriminant validity were lower than the proposed limit of 0.85 (Gold et al., 2001; Henseler et al., 2015; Teo et al., 2008), suggesting that the three latent variables utilised in the study were conceptually distinct.

**Table 4:** Discriminant validity (Fornell-Larcker and Heterotrait-Monotrait Criteria).

	Fornell-Larcker Criterion			Heterotrait-Monotrait Ratio (HTMT)		
	PCS	JS	JI	PCS	JS	JI
Perceived co-worker support (PCS)	0.879					
Job stress (JS)	−0.208	0.747		0.214		
Job involvement (JI)	0.399	−0.201	0.858	0.507	0.256	-

### 4.2 Model estimation

The standard root mean square residual (SMSR) value was used to evaluate the model fit (Henseler et al., 2016). The SRMR of the model was 0.066 < 0.08, indicating a good model fit (Hu and Bentler, 1998) (Table 5). The models' explanatory power was assessed using the adjusted *R*^2^ criterion (Shmueli and Koppius, 2011). The result from the study shows the combined effect of job stress and co-worker support explains 35.6% of the variations in interns' job involvement. Stone-Geisser’s *Q*^2^ Test (Geisser, 1974; Stone, 1977) was used to assess the predictive validity of the model. The Q^2^ values of job involvement (0.128) demonstrate medium predictive relevance (Hair et al., 2019). Cohen's (1988) *f*^*2*^ was used to assess the effect size of the exogenous construct. Analysis of the results suggests the magnitude of the effect of internship work stress on job involvement (*f*^*2*^ *=* 0.124) threshold of medium effect size.

**Table 5:** Summary of fit and R^2^ of structural model.

Construct Coefficient of Determination (R^2^)	R^2^	Adjusted R^2^
Job involvement	0.361	0.356
Model Fit	Value	
SRMR	0.066	

Prior to the testing of hypotheses, the collinearity between the predictor variables was evaluated using a variance inflation factor (VIF) (Hair et al., 2016). As a rule of thumb, collinearity is absent if the VIF value is less than 3. Table 6 shows that the VIF values of the pairs of internship work stress and co-worker support are all below 3, indicating that there is no collinearity between these two predictors of job involvement.

**Table 6:** Collinearity assessment (inner VIF values).

	PCS	JS	JI
Perceived co-worker support (PCS)		1.050	
Job stress (JS)		1.111	
Job Involvement (JI)			

The results of direct (H1) and moderating (H2) hypotheses as shown in Table 7 reveal that the *p*-values of the 2 paths estimated were significant.

**Table 7:** Assessment of direct and indirect hypotheses.

Hyp.	Path	Path Coefficient	*t* Statistics	*p* Values
H1	Job stress - > Job involvement	−0.930	14.652	0.000
	*Moderating effect of perceived co-worker support on*			
H2	Job stress - > Job involvement	0.231	0.612	0.000

H1 was confirmed as the relation between job stress and job involvement was negative and significant (*β* = −0.930; *t*-value = 14.652; *p* = 0.000). The finding suggests students' involvement is not feasible when they experience excessive job stress during their internship.

**Figure 2:**
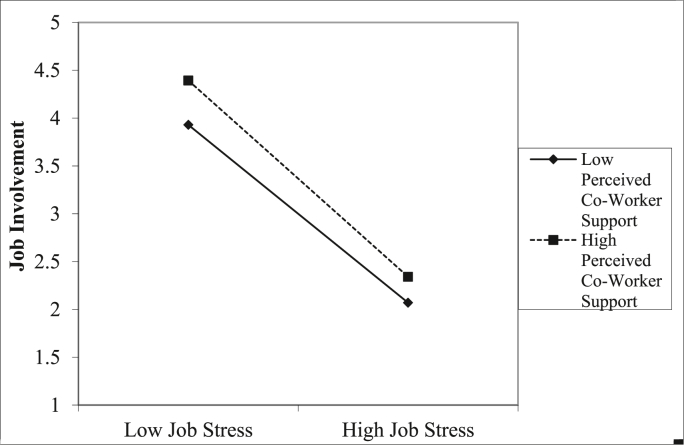
Simple Slope of perceived coworker support on job stress and job involvement.

H2 was confirmed as the moderation of the relation between job stress and job involvement by perceived co-worker support was positive and significant (*β* = 0.231; *t*-value = 0.612; *p* = 0.000). This suggests the relationship between interns' job stress and job involvement is strengthen by the presence of perceived co-worker support. Finally, the study examined simple slopes at low and higher levels of co-worker support on the expected relationship between job stress and job involvement. The plot is illustrated in Figure 2. The results suggest that interns' job involvement is higher for lower job stress and lower in the face of higher job stress. Additionally, the relationship between job stress and job involvement is stronger for interns who perceive higher co-worker support (R = .531, p < 0.001) compared to interns who perceive lower co-worker support (R = .342, p < 0.012).

## 5 Discussion

In this study, we address two main objectives: (i) the influence of job stress on intern’s job involvement and (ii) the moderating influence of perceived co-worker support on the nexus between intern’s job stress and job involvement. Consistent with prior studies (Adekeye et al., 2017; Demir, 2018; Li, 2018; Walia and Narang, 2015), job stress negatively predicts intern’s job involvement, thereby supporting H1. This study adds to the existing literature on the influence of job stress on job involvement from the perspective of interns in the higher education environment. Under conditions where interns are not paid, and the level of stress is extremely high, such students are compelled to reduce their level of job involvement. The finding is plausible because the phenomenon may likely reduce intern’s efficiency, diminish their fulfilment and consequently, decrease their commitment to pursue a career in their chosen fields of study.

In addition, perceived co-worker support mitigates the negative association between interns' job stress and job involvement, thereby supporting H2. This finding corroborates earlier studies that found perceived co-worker support as a useful boundary conditioning (Rehman et al., 2019; Robertson et al., 2016; Shin et al., 2021). The positive moderation of the association between job stress and job involvement is reasonable since co-worker’s act as confidants, ease workloads, and make harsh work situations more bearable (López et al., 2019). Furthermore, co-workers’ support enriches interns' work experience and reduces the harmful effects of unfair treatment (Sloan, 2012). This finding is noteworthy since it’s the first time a positive moderating impact of perceived co-worker support has been found in a direct relation between job stress and job involvement in the context of student internship studies. The findings suggest the easy and early acclimatization of interns at the work environment hinges on the relations and the extent to which full-time employees are willing help students. Given the critical role of co-workers, it is reasonable to conclude that uncooperative behaviour of full-time staff may likely affect the ability of students to achieve their learning objectives and further exacerbate the stress experience of interns thereby reducing their level of job involvement.

### 5.1 Implications for theory and practice

Theoretically, stress has been shown to negatively predict job involvement among university interns, a relationship that remains unexplored in the higher education literature. Furthermore, the empirical finding demonstrates how co-worker support enhances intern job involvement. The study’s model elucidates the theoretical concept that when interns are assisted by full-time employees, they acquire a positive attitude towards work by becoming more involved in their job. These findings add to the body of knowledge on internships by demonstrating how perceived co-worker support has a substantial impact on the link between stress and the intern’s job involvement.

The results have far-reaching ramifications for internships for students. Fortunately, strong support from co-workers was found to improve interns' job involvement. Consequently, organisations must build a supportive work culture to improve interns' job involvement. Ultimately, intervention relating to co-worker support should be encouraged at the workplace during students' internships. This would make interns feel more confident and capable of completing their responsibilities. This assistance might take the form of knowledge, emotional support, or positive reinforcement. For example, based on informational support, co-workers can meet frequently with interns where they talk about the work unit’s demands and problems and get help in devising workable solutions to any task-related problems that occur. On constructive feedback, co-workers can support interns by using performance reviews to provide constructive practical advice and guidance on areas that need improvement. Additional efforts must be made to reduce the stress levels of interns. For example, higher educational institutions should organise induction training for interns to psychologically prepare them as well as refine their knowledge and abilities before exposing them to real-work conditions. Furthermore, more institutions must establish a pre-internship programme in which students are educated about the workplace. Furthermore, these programmes must contain methods that encourage students to develop resilience. Organizations must put in place intervention programmes aimed at reducing intern stress at work. For example, the organisation could reduce intern stress by allocating more time and resources to specific tasks and (ii) increasing employee engagement in work planning and decision-making.

### 5.2 Limitations and future research directions

First, the survey respondents had varied years of internship experience and worked for either a private or public organisation throughout their internship. These two circumstances may have an influence on the conclusion since the experiences of interns differ. Future studies may address this problem by collecting data from a sample based on type of organisation and the duration of the internship.

The outcomes of the study hint at several future research prospects. The focus of this study was on unpaid internships, which are widespread among students in the context where the study was conducted. Future studies can extend the study in the context of paid internships. Future research could also investigate other aspects of work-based support, such as supervisory, social, and organisational support, as moderators in the relationship between internship stress and job engagement. Third, the research model was examined at the individual level. The idea should be re-tested at the team level in future investigations. Finally, this model may be tested in various scenarios for validation reasons.

## Declarations

### Author contributions statement

Edem M. Azila-Gbettor: conceived and designed the experiments; performed the experiments; analyzed and interpreted the data; wrote the paper.

Ernestina Atsu: Performed the experiments; Contributed reagents, materials, analysis tools or data; wrote the paper.

Abigail Nana Konadu Quarshie: Performed the experiments; Contributed reagents, materials, analysis tools or data; wrote the paper.

### Funding statement

This research did not receive any specific grant from funding agencies in the public, commercial, or not-for-profit sectors.

### Data availability statement

Data will be made available on request.

### Declaration of interests statement

The authors declare no conflict of interest.

### Additional information

Supplementary content related to this article has been published online at https://doi.org/10.1016/j.heliyon.2022.e10414.

## Appendix A Supplementary data

Questionnaires Questionnaires
