# Supplementary material for: Job stress and job involvement among tertiary interns: the buffering role of perceived coworker support
Source: Heliyon. 2022 Aug 30;8(9):e10414. doi: 10.1016/j.heliyon.2022.e10414 (PMC9469660; doi:10.1016/j.heliyon.2022.e10414)
Supplement: Questionnaires [file mmc1.docx]

***Perceived Co-worker Support***

1. Workers at the workplace were friendly to me
2. Workers at the workplace were helpful to me
3. Workers were interested in me
4. Workers at the workplace place were competent
5. Workers at the workplace place always encouraged me
6. Workers were helpful in getting my job done
7. Workers gave useful advice on job problems
8. Workers took over my work/task anytime I was tired

***Job stress***

1. My job is very complicated, and there is a heavy workload
2. Worry about personal safety at job
3. I often job overtime in my job
4. Great responsibility, afraid of accountability
5. I do not know much about my job
6. My job has not been clearly explained and explained
7. Sometimes I receive different job requirements from the job leaders
8. Sometimes I am assigned to different positions at the same time
9. Conflict or unhappiness with colleagues
10. Feel isolated at the job
11. Lack of support from leadership
12. Leaders were unwilling or unable to help me with my job problems
13. The organization did not respond well to my performance
14. I am worried about my future career development
15. My rights are sometimes not protected

***Job involvement***

1. My job is the most important part of my life
2. I do not feel emotionally involved in my job
3. I would feel guilty if I left my days job incomplete
4. I do not enjoy my job
